# Differential miRNA expression in B cells is associated with inter-individual differences in humoral immune response to measles vaccination

**DOI:** 10.1371/journal.pone.0191812

**Published:** 2018-01-30

**Authors:** Iana H. Haralambieva, Richard B. Kennedy, Whitney L. Simon, Krista M. Goergen, Diane E. Grill, Inna G. Ovsyannikova, Gregory A. Poland

**Affiliations:** 1 Mayo Clinic Vaccine Research Group, Mayo Clinic, Rochester, Minnesota, United States of America; 2 Department of Health Sciences Research, Mayo Clinic, Rochester, Minnesota, United States of America; Universitat des Saarlandes, GERMANY

## Abstract

**Background:**

MicroRNAs are important mediators of post-transcriptional regulation of gene expression through RNA degradation and translational repression, and are emerging biomarkers of immune system activation/response after vaccination.

**Methods:**

We performed Next Generation Sequencing (mRNA-Seq) of intracellular miRNAs in measles virus-stimulated B and CD4^+^ T cells from high and low antibody responders to measles vaccine. Negative binomial generalized estimating equation (GEE) models were used for miRNA assessment and the DIANA tool was used for gene/target prediction and pathway enrichment analysis.

**Results:**

We identified a set of B cell-specific miRNAs (e.g., miR-151a-5p, miR-223, miR-29, miR-15a-5p, miR-199a-3p, miR-103a, and miR-15a/16 cluster) and biological processes/pathways, including regulation of adherens junction proteins, Fc-receptor signaling pathway, phosphatidylinositol-mediated signaling pathway, growth factor signaling pathway/pathways, transcriptional regulation, apoptosis and virus-related processes, significantly associated with neutralizing antibody titers after measles vaccination. No CD4^+^ T cell-specific miRNA expression differences between high and low antibody responders were found.

**Conclusion:**

Our study demonstrates that miRNA expression directly or indirectly influences humoral immunity to measles vaccination and suggests that B cell-specific miRNAs may serve as useful predictive biomarkers of vaccine humoral immune response.

## Introduction

Immune responses to vaccines (e.g., live measles vaccine) have a high degree of inter-individual variation, including poor/non-response, which eventually leads to an accumulation of susceptible individuals and subsequent disease outbreaks. Studies focusing on host genetic factors, transcriptional responses (differential gene expression upon measles virus exposure or *in vitro* viral stimulation), environmental, demographic and clinical variables, have tried to elucidate the mechanisms behind these variations in measles antibody titers. [1,2,3,4,5,6] Multiple studies in our laboratory have led to estimates that genetic factors (HLA alleles and common single nucleotide polymorphisms) only explain ~30% of the inter-individual variation in antibody titers after measles vaccination.[1] Novel high-dimensional technologies, omics assays, and vaccinomics/systems biology approaches [1] are increasingly being applied to vaccine studies in order to identify other biomarkers of protective and non-protective (low) vaccine-induced immune responses.

MicroRNAs (miRNA) have emerged as master regulators of RNA silencing and post-transcriptional modulation of gene expression. Over 1,000 miRNA species have been identified in the human genome, targeting more than 60% of the human genes. [7] miRNAs are small non-coding (21 to 23 nucleotides) sequences encoded in the intergenic regions or within introns/exons of genes. Functional miRNAs are produced from larger pre-miRNA transcripts (hairpins) cleaved in the cytoplasm by the endonuclease enzyme Dicer. One-strand miRNAs are assembled into the RNA-induced silencing complex (RISC), which binds to 3’ UTR sequences of target mRNA molecules with a sequence specificity provided by the miRNA component of the complex. This process results in RNA silencing through target mRNA cleavage, destabilization, or lower translation efficiency [8]. Each miRNA is able to control multiple (sometimes inter-related) genes and thus represents an important regulatory feature of the transcriptome. Dysregulated miRNA expression has been associated with various diseases and biological processes, including autoimmunity, and infectious diseases, immune function and viral replication. [8,9,10,11,12]

Expert opinion and the literature supports the importance of humoral immunity and neutralizing antibodies in protection against measles, and antibody titers above 120–200 mIU/ml have been accepted as a correlate of protection.[1, 2, 3, 4, 5, 6, 13, 14] Antigen-induced B cell differentiation with the help of CD4^+^ T cells is recognized as a key biological phenomenon underlying the formation of ASCs (and memory B cells) to mount a protective anti-viral humoral immune response. [13,14].

The goal of the current study was to characterize B and T-cell-specific miRNA profiles after *in vitro* measles virus stimulation in order to identify distinctive miRNAs—along with their targeted genes and pathways—that are associated with high (protective) and low (below the level of protection) neutralizing antibody titers following measles vaccination.

## Methods

The methods described here are similar or identical to our previously published papers involving the described methodology and this cohort. [3,6,15,16,17,18,19,20]

### Study subjects

Twenty-three subjects were selected for miRNA profiling from previously recruited subjects (3,191 healthy children/adolescents and young adults; 11–40 years old) who received two doses of MMRII vaccine. The demographic, clinical, and immune variables of this large cohort have been previously published. [6,15] The subjects for the current study were selected based on sample availability and extremes of neutralizing antibody titer (11 high antibody responders with a median titer of 2,055 mIU/mL, and 12 low antibody responders with a median titer of 246 mIU/mL). The currently accepted threshold for protection against symptomatic measles infection is a plaque reduction neutralization titer ≥120, corresponding to 210 mIU/mL in this study.[18]

The Institutional Review Boards of the Mayo Clinic (Rochester, MN) and the Naval Health Research Center (NHRC) (San Diego, CA) approved the study, and written informed consent was obtained from each subject (i.e., from age-appropriate participants, and the parents of all children who participated in the study).

#### Plaque reduction microneutralization assay (PRMN)

As previously published, measles virus (MV)-specific neutralizing antibody titers were measured using a high-throughput plaque-reduction microneutralization fluorescence-based assay with a coefficient of variation in our laboratory of 5.7%. [3,18] This assay quantifies all anti-measles virus neutralizing antibodies (i.e., both anti-H and anti-F antibodies). Karber’s formula was used to calculate the 50% neutralizing dose (ND_50_) and transformed into mIU/mL using the third international WHO anti-measles antibody standard. [18]

### Cell purification and miRNA isolation

Cryopreserved PBMCs were thawed using standard procedures.[21] B cells and monocytes were purified using a human CD19 MicroBeads positive selection kit (Miltenyi Biotec; San Diego, CA) and a human CD14 MicroBeads positive selection kit (Miltenyi Biotec), respectively. B cells and monocytes cells were left uninfected, or were infected overnight with the Edmonston strain of MV at a multiplicity of infection 0.2. CD4^+^ T cells were isolated using the CD4^+^T Cell Isolation Kit (Miltenyi Biotec) and co-cultured overnight with or without MV-infected monocytes, after which the CD4^+^ T cell isolation was repeated (on the mixed cultures) to ensure that the T cell samples were not contaminated with monocytes. The purity of the cells was ascertained using flow cytometry. Total RNA (including miRNA) from B and T cells was extracted using miRNeasy Mini Kits (Qiagen; Valencia, CA) according to manufacturer’s instructions.

### Next Generation Sequencing and Bioinformatics

Subjects were randomized prior to library preparation. Three subjects, each with four cell types/samples (unstimulated B cells, stimulated B cells, unstimulated CD4^+^ T cells and stimulated CD4^+^ T cells), were allocated to a single lane; therefore, lanes were balanced by cell type in addition to immune response status and sex. The quantity and quality of extracted RNA were assessed using an Agilent 2010 Bioanalyzer (Agilent; Palo Alto, CA). Illumina TruSeq miRNA library construction was performed at the Mayo Clinic Advanced Genomics Technology Center to generate small RNA libraries. Adapter sequences were ligated onto the 3' and 5' ends of each miRNA, and then the miRNA products were PCR-enriched and purified. Library validation and quantification was carried out using DNA 1000 Nano Chip kits on an Agilent 2100 Bioanalyzer (Agilent; Palo Alto, CA). Twelve samples were multiplexed in each lane. Libraries were loaded onto flow cell lanes and single-end read sequencing was performed using the Illumin HiSeq 2000 (Illumina; San Diego, CA), as described previously [3,6,22].

The Mayo Clinic Bioinformatics Systems Unit processed the FASTQ files using a comprehensive pre-processing and analytical pipeline (CAP-miRNA)[23] that assesses read quality (FastQC), trims adapter sequences (Cutadapt), and aligns reads for IGV viewing and detection of mature, precursor, and novel miRNAs (miRDeep2).[24]

### Statistical analysis

B-cell samples and CD4^+^ T-cell samples were evaluated separately. Prior to normalization, mature miRNA was filtered using a median cutoff of 4, leaving 351 miRNAs available for the analysis of B-cell samples and 339 miRNAs for the analysis of CD4^+^ T-cell samples. Normalization was performed using the trimmed mean of M-values (TMM) normalization method [25] and was evaluated visually using boxplots and minus-versus-average plots. One CD4^+^ T-cell sample failed quality control and was removed from further analysis. Negative Binomial Generalized Estimating Equation (GEE) models [26], comparing stimulated versus unstimulated samples and stimulated high responders versus stimulated low responders, were run for each miRNA. Models were run in SAS using the proc genmod procedure, incorporating the TMM offset and Tagwise dispersion parameters estimates that were computed in R using the edgeR package.[27] Q-values were computed using the Storey and Tibshirani method (2003).[28]

The top miRNA results from the GEE models (q-value < 0.10) for B-cell interaction analysis (n = 14), B-cell overall analysis (n = 8), and CD4^+^ T-cell overall analysis (n = 15, cutoff at top 15 miRNA) were assessed using the DIANA pathway tool.[29] We analyzed Genes Union and Categories/Pathways Union methods for both KEGG and GO pathways using the default settings.

## Results

### Study subjects’ characterization

For this study, we used samples from 23 subjects representing the extremes of the neutralizing antibody response after measles vaccination (following two doses of MMR II vaccine) from a large study cohort. The median neutralizing antibody titer of the high antibody responders (n = 11) was 2,055 mIU/mL, while the median neutralizing antibody titer of the low antibody responders (n = 12) was 246 mIU/mL. All demographic and clinical/immune variables are presented in Table 1. Despite differences in vaccine-induced antibody titer, no statistically significant differences in other variables (including secretion of MV-specific Th1/Th2 and innate/inflammatory cytokines; and MV-specific IFNγ ELISPOT response) were noted between the two responder groups (Table 1).

**Table 1:** Demographic and immune response variables of the study subject.

	Low Ab responders (n = 12)	High Ab responders (n = 11)	Total (n = 23)	P-value^b^
**Age at enrollment, years**				0.223
Median (IQR)^a^	14.5 (12.8, 18.2)	13 (12, 14)	13 (12, 16)	
**Age at 1**^**st**^ **measles immunization, months**				0.317
Median (IQR)^a^	15 (15, 17.2)	15 (13.5, 15.8)	15 (14.5, 16)	
**Age at 2**^**nd**^ **measles immunization, years**				0.598
Median (IQR)^a^	5 (4.25, 9.5)	5 (4, 5)	5 (4, 5)	
**Time from 2**^**nd**^ **immunization to enrollment, years**				0.641
Median (IQR)^a^	7.85 (6.25, 9.05)	8.4 (7.45, 8.7)	8.3 (6.7, 8.8)	
**Sex**				1.000
Female	5 (41.7%)	5 (45.5%)	10 (43.5%)	
Male	7 (58.3%)	6 (54.5%)	13 (56.5%)	
**Race**				0.364
White	11 (91.7%)	7 (63.6%)	18 (78.3%)	
Black or African American	1 (8.33%)	2 (18.2%)	3 (13%)	
Multiple/Other	0 (0%)	2 (18.2%)	2 (8.7%)	
**Ethnicity**				0.936
Not Hispanic or Latino	11 (91.7%)	9 (81.8%)	20 (87%)	
Hispanic or Latino	1 (8.33%)	2 (18.2%)	3 (13%)	
**Neutralizing antibody, mIU/mL**				NA^c^
Median (IQR)^a^	246 (203, 322)	2055 (1960, 2937)	391 (246, 2025)	
**IL-2, pg/mL**				0.511
Median (IQR)^a^	52.1 (32.5, 69)	44.3 (20, 70.5)	52.1 (24.2, 71.5)	
**IFNγ, pg/mL**				0.516
Median (IQR)^a^	68.3 (37.3, 149)	36.9 (12.9, 162)	58.6 (21, 165)	
**IL-10, pg/mL**				0.407
Median (IQR)^a^	29.7 (19.5, 48.9)	19.2 (15.9, 26)	20.6 (18.5, 41.3)	
**IL-6, pg/mL**				0.079
Median (IQR)^a^	385 (350, 475)	271 (226, 349)	350 (246, 429)	
**TNFα**				0.203
Median (IQR)^a^	8.15 (6.85, 15.1)	16.2 (13.2, 17.6)	14.6 (7.34, 17)	
**IFNα**				0.570
Median (IQR)^a^	689 (376, 1384)	695 (113, 1128)	689 (254, 1305)	
**IFNλ1**				0.885
Median (IQR)^a^	46.1 (30.9, 90.5)	67.7 (7.24, 117)	49.1 (18.9, 112)	
**IFNγ ELISPOT, SFU / 2x10**^**5**^ **cells**^d^				0.598
Median (IQR)	29 (13.2, 37.5)	24 (13.5, 33)	26 (11.5, 36)	

^a^IQR,25% and 75% inter-quartile range

^b^P-values are calculated using Wilcoxon Rank Sum test

^c^Not applicable since subjects were selected based on antibody titer

^d^Spot-forming units per 200,000 cells

### miRNA expression in B and CD4^+^ T cells after in vitro MV stimulation

We first assessed miRNA expression in purified MV-stimulated B and CD4^+^ T cells irrespective of immune response status to measles vaccination (i.e., overall analysis in all samples to assess the effect of viral stimulation). The results (see S1 and S2 Tables) identify 10 and 329 miRNAs (q-value <0.2) for B and CD4^+^ T cells, respectively, that were significantly up/down-regulated upon MV stimulation. We observed five overlapping miRNAs that were significantly regulated in both B and CD4^+^ T cells (i.e., hsa-miR-409-3p, hsa-miR-543, hsa-miR-10b-5p, hsa-miR-7704, and hsa-miR-99b-5p). These differentially expressed miRNAs were found to regulate many common (for B and CD4^+^ T cells) biological pathways and processes, such as several signaling pathways, viral infection-associated processes, and processes related to regulation of transcriptional activity (S3 Table). In addition, some miRNAs were also found to regulate unique (for the examined cell subset) pathways/processes; for example, Toll-like receptors (TLRs) and TLR signaling pathway genes that were targeted by miRNAs in B cells after MV stimulation (S3 Table).

### Specific B cell miRNAs are associated with neutralizing antibody titers after measles vaccination

We also evaluated lymphocyte-specific miRNAs demonstrating differences in expression between high and low antibody responders to measles vaccination (q-value < 0.2). Per-miRNA negative binomial GEE modeling was utilized to identify B cell-specific (and T cell-specific) miRNAs discriminating high from low antibody response. This yielded a group of 21 statistically significant miRNAs from B cells that were correlated with measles-specific neutralizing antibody titer after vaccination (p<0.017, q-value <0.2, Table 2). Although we found differential miRNA expression in CD4^+^ T cells upon MV stimulation (i.e., overall analysis in all samples), no CD4^+^ T cell-specific miRNA expression differences between high and low antibody responders were noted when we compared the two groups (q-value <0.2, S4 Table).

**Table 2:** B cell-specific miRNAs demonstrating differences in expression between high and low antibody responders to measles vaccination (interaction analysis, q-value < 0.2).

miRNA	FC^a^	Log2FC	Std.Err.Log2FC	p-value	q-value
hsa-miR-151a-5p	2.88	1.53	0.37	3.94E-05	0.008
hsa-miR-107	2.75	1.46	0.37	6.83E-05	0.008
hsa-miR-15a-5p	3.80	1.93	0.51	0.0002	0.013
hsa-miR-3690	4.64	2.21	0.61	0.0003	0.015
hsa-miR-20b-5p	3.82	1.93	0.57	0.0006	0.029
hsa-miR-421	4.27	2.09	0.67	0.002	0.063
hsa-miR-16-5p	2.87	1.52	0.49	0.002	0.063
hsa-miR-103a-3p	2.98	1.57	0.52	0.003	0.073
hsa-miR-199a-3p	2.10	1.07	0.36	0.003	0.074
hsa-miR-223-3p	4.40	2.14	0.74	0.004	0.080
hsa-miR-221-3p	2.64	1.40	0.49	0.004	0.080
hsa-miR-30b-5p	3.71	1.89	0.66	0.004	0.080
hsa-let-7g-5p	1.95	0.96	0.34	0.005	0.080
hsa-miR-185-5p	2.31	1.21	0.43	0.005	0.080
hsa-miR-98-5p	1.96	0.97	0.36	0.007	0.103
hsa-miR-26b-5p	2.32	1.22	0.46	0.008	0.113
hsa-miR-93-5p	2.88	1.52	0.58	0.008	0.113
hsa-miR-29b-3p	2.40	1.27	0.49	0.010	0.125
hsa-miR-151b	2.04	1.03	0.41	0.013	0.153
hsa-miR-502-3p	2.29	1.20	0.50	0.016	0.180
hsa-miR-29c-3p	2.04	1.03	0.43	0.017	0.180

^a^Fold change of interaction

### miRNA-modulated biological processes and pathways associated with neutralizing antibody response in high vs. low antibody responders to measles vaccination

Predicted targets of the B cell-specific miRNAs (demonstrating differences between high and low antibody responders) were identified (for miRNAs with q-value < 0.10, Table 2). Pathway-enrichment analysis identified GO/KEGG pathways, including Fc-receptor and several other signaling pathways, as well as pathways related to transcriptional regulation, viral infection, lipid biosynthesis/metabolism, cytoskeletal protein binding, extracellular matrix (ECM)-receptor interactions and apoptosis. The activity of those pathways is likely differentially regulated by miRNAs in B cells of high and low antibody vaccine responders (Table 3).

**Table 3:** Immune function-related pathways/biological processes differentially regulated by MV response-specific miRNAs in B cells of high and low antibody vaccine responders.

Pathway	P-value	#Genes	#miRNAs
Cellular nitrogen compound metabolic process	<1.0E-325	1154	12
Neurotrophin TRK receptor signaling pathway	<1.0E-325	88	9
Fc-receptor signaling pathway	<1.0E-325	59	9
Viral process	<1.0E-325	95	7
Fatty acid biosynthesis	<1.0E-325	4	4
Fatty acid metabolism	<1.0E-325	8	4
Epidermal growth factor receptor signaling pathway	1.11E-16	48	6
Signaling pathways regulating pluripotency of stem cells	2.01E-14	37	5
Nucleic acid binding transcription factor activity	3.71E-14	236	8
Extracellular matrix (ECM)-receptor interaction	4.32E-13	12	2
Blood coagulation	4.6E-13	110	8
Fibroblast growth factor receptor signaling pathway	3.1E-11	40	5
Protein binding transcription factor activity	7.16E-09	101	5
Cell death	1.18E-08	182	7
Cytoskeletal protein binding	1.22E-08	133	4
Phosphatidylinositol-mediated signaling	2.56E-08	36	5
Mucin type O-Glycan biosynthesis	7.44E-07	10	4

## Discussion

MicroRNAs are important mediators of post-transcriptional regulation of gene expression through RNA degradation, translational repression, and other mechanisms. They have been demonstrated to regulate various immune functions and are plausible biomarkers of lymphocyte activation and infectious diseases pathogenesis, course and clinical outcome. [30,31,32,33] Several studies have provided evidence for altered miRNA expression in humans infected with influenza A/H1N1 and A/H3N2 viruses, HIV-1, VZV, HBV, and HCV. [33,34,35,36,37] Given the critical role of host miRNAs in the regulation of gene expression, antiviral response and immunity, miRNAs are emerging as important markers of responsiveness to vaccination (e.g., influenza and hepatitis B vaccination).[38, 39, 40] While we and others have identified specific gene-expression patterns during measles infection and/or vaccination and identified (gene) transcriptional signatures associated with antibody response after measles vaccination [3,4,5], intracellular and/or serum miRNAs have never been explored as factors influencing measles immunity. The results of the current study demonstrate that intracellular B cell-specific miRNA expression is associated with direct or indirect regulation of humoral immunity to measles vaccination.

The assessment of MV-stimulated miRNA expression in purified B and CD4^+^ T cells from vaccinees revealed several pathways/biological processes targeted by miRNAs in both cell types (e.g., transcriptional regulation, TGF-beta signaling pathway, epidermal and fibroblast growth factor receptor-signaling pathways, neurotrophin TRK receptor-signaling pathway, extracellular matrix (ECM)-receptor interactions, and viral infection processes). TGF-beta signaling pathway, for example, is an essential pathway in the maintenance of T and B cell homeostasis. It regulates T helper cell differentiation, the development of central memory, the response to antigenic stimulation, and is vital for regulatory T cell function. This pathway may also directly impact B cell expansion/homeostasis, immunoglobulin concentrations and antigen presentation.[41, 42] Thus, the observed MV-stimulated expression of miRNAs targeting this pathway in both B and CD4^+^ T cells of vaccinees is likely to impact adaptive immune function, although no miRNA expression differences (of these specific miRNAs) were found between the high and low antibody responder groups in our study. Of note, we observed virus-induced differential expression of miRNAs affecting multiple TLR signaling pathways in B cells, including the TLR2 signaling pathway, which is known to be activated by the MV hemagglutinin. [43]

Evaluation of intracellular miRNA expression differences between the high and the low antibody responder groups enabled us to identify of a set of B cell-specific miRNAs that were associated with neutralizing antibody response after measles vaccination (i.e., they were differentially expressed after MV stimulation in the B cells of high vs. low antibody responders). The top miRNA, miR-151a-5p, among other targets, regulates the expression of *MT-CYB* (mitochondrially encoded cytochrome b) and thus may regulate critical cellular function, such as the mitochondrial respiratory activity and ATP production. [44] Predicted gene targets of miR-151a-5p include *NXF1* (involved in the regulation of gene expression of MHC class II molecules), *TAPBP* (TAP-binding protein involved in the assembly of MHC class I complex), *RHOH* (expressed in hematopoietic cells, regulator of cell growth and survival), *EEF2* (the eukaryotic translation elongation factor 2), and five genes of the adherens junctions pathway (*ACTB*, *IQGAP1*, *CTNNB1*, *INSR*, and *MAPK1*). Although the adherens junction protein nectin-4 (*NECTIN4/PVRL4*), which is a recently identified epithelial receptor for MV [45,46], was not found to be regulated by the identified set of miRNAs, it is likely that adherens junction proteins, extracellular matrix-receptor interactions (highlighted pathway in Table 3), and the adhesion and regulation of the cytoskeleton are important biological processes involved in MV infection and measles immunity. In support of this, three other B-cell miRNAs associated with vaccine-induced antibody titers (Table 2) were found to target the gene expression of nectins (miR-15a-5p of *PVRL1*, *PVRL2* and *PVRL3*, miR-199a-3p of *PVRL3*, and miR-103a-3p of *PVRL1* and *PVRL2*). The differential regulation of miR199a, for example, has been reported during hepatitis C infection and is associated with pathogenicity, and in addition, miR199a has been found to inhibit viral replication of some herpesviruses and alphaviruses. [33,34,35,36,37,47] Other interesting miRNAs include the following: miR-223, which is implicated primarily in the regulation of granulocyte, erythrocyte, and NK-cell development/function (e.g., *GZMB* is a direct target of miR-223) and in HIV-1 infection [48,49,50]; the miR-15a/16 cluster, which targets pro-apoptotic genes and apoptosis regulators (*BCL2*) that are key for cell-cycle regulation and apoptosis in B cells [51,52]; and miR-29/29c, a critical factor in the regulation of virus-induced apoptosis and innate immunity to influenza A/H1N1 and A/H3N2 through targeting *BCL2L2*, *DNMT*, *COX2* activity, IFN-alpha receptor (*IFNAR1*) and the Jak/STAT signaling pathway. [35,47,53] Mir-29 has also been reported to target and downregulate the viral Nef protein expression during HIV-1 infection. [33,34,35,36,37] In addition, enriched pathways and processes with relevance to adaptive immunity (that are regulated by the identified set of B-cell miRNAs) include the Fc-receptor signaling pathway, the phosphatidylinositol-mediated signaling pathway, growth factor signaling pathways, transcriptional regulation activity, apoptosis, metabolism, and virus-related processes. The differential activation of signaling pathways by different Fc receptors, for example, is known to promote a wide variety of immune cellular functions and is closely involved in immune regulation. [54]

The strengths of our study include the use of purified cell populations from subjects with the extremes of antibody response (high and low antibody response to measles vaccine) and the use of a new technology/analysis not previously applied to measles (first miRNA study on measles vaccine) to assess immunity after vaccination. Limitations include the relatively small sample size (n = 23) and the lack of replication and/or functional validation of identified gene targets and pathways that warrant further exploration. Further functional studies (e.g., RISC-Seq) are needed to identify/validate suggested miRNA targets and the cellular processes and functions regulated by vaccine/virus-induced miRNAs. [55] The overall assessment of miRNA expression in MV-stimulated T and B cells irrespective of immune response status (overall analysis, S1 and S2 Tables) yielded the identification of more T cell-specific miRNAs than B cell-specific miRNAs. However, as noted, no CD4^+^ T cell-specific miRNA expression differences between high and low antibody responders were found (i.e., only B cell-specific miRNA expression differences were identified) when we compared the two groups. This could be related to the relatively small sample size and/or to the fact that the groups were selected based on antibody response (and not MV-specific cellular immune response), which is directly related to biological processes and pathways regulated in B cells. To differentiate the measles virus-specific miRNA expression from the miRNA expression specific to other components of the MMR vaccine (i.e., mumps and rubella), we used measles virus *in vitro* stimulation of purified B and T cells from vaccinees. While this study design may not fully recapitulate miRNA regulation *in vivo* after MMR vaccination, no practical method exists to administer measles vaccine alone in the US. Regardless the listed limitations, the biologic plausibility of the identified targets and pathways in regards to humoral immunity, and the lack of any other pre-existing studies in this area, all increase the importance of disseminating these findings.

In conclusion, our study provides new knowledge on vaccine-induced immune function and modulation of immune response via miRNA regulation of gene expression. We identified B cell-specific miRNAs that are likely regulating humoral immunity after measles vaccination, which may explain (in part) inter-individual variations in antibody response. These miRNAs, in concert with their gene targets and/or other measles-specific transcriptomic [3,18] and immunogenetic [1,2,3,4,5,6] signatures, could potentially serve as predictive biomarkers of measles vaccine antibody response.

## Supporting information

S1 Table miRNA expression in MV-stimulated B cells irrespective of immune response status (i.e., overall analysis in all samples, q<0.2). (DOCX)

S2 Table miRNA expression in MV-stimulated CD4^+^ T cells irrespective of immune response status (i.e., overall analysis in all samples, q<0.2). (DOCX)

S3 Table Pathways and biological processes differentially regulated upon stimulation with MV in B cells or CD4^+^ T cells (overall analysis in all samples). (DOCX)

S4 Table CD4^+^ T cell-specific miRNA expression differences between high and low antibody responders to measles vaccination (no significant differences were noted, top 10 miRNAs are listed). (DOCX)
