# Supplementary material for: Differential miRNA expression in B cells is associated with inter-individual differences in humoral immune response to measles vaccination
Source: PLoS One. 2018 Jan 30;13(1):e0191812. doi: 10.1371/journal.pone.0191812 (PMC5790242; doi:10.1371/journal.pone.0191812)
Supplement: S1 Table — (DOCX) [file pone.0191812.s001.docx]

**Table S1** miRNA expression in MV-stimulated B cells irrespective of immune response status (i.e., overall analysis in all samples, q<0.2)

| **miRNA** | **FC** | **Log2FC** | **Std.Err.Log2FC** | **p-value** | **q-value** |
| --- | --- | --- | --- | --- | --- |
| hsa-miR-409-3p | 3.225 | 1.689 | 0.224 | 4.48E-14 | 1.32E-11 |
| hsa-miR-543 | 3.452 | 1.787 | 0.249 | 7.1E-13 | 7.36E-11 |
| hsa-miR-134-5p | 8.438 | 3.077 | 0.429 | 7.51E-13 | 7.36E-11 |
| hsa-miR-381-3p | 8.922 | 3.157 | 0.490 | 1.13E-10 | 8.26E-09 |
| hsa-miR-485-5p | 2.789 | 1.480 | 0.235 | 3.28E-10 | 1.93E-08 |
| hsa-miR-10b-5p | 2.662 | 1.412 | 0.256 | 3.44E-08 | 1.68E-06 |
| hsa-miR-184 | 0.448 | -1.159 | 0.294 | 8.27E-05 | 0.003 |
| hsa-miR-4746-5p | 0.533 | -0.909 | 0.291 | 0.002 | 0.065 |
| hsa-miR-7704 | 0.637 | -0.650 | 0.220 | 0.003 | 0.102 |
| hsa-miR-99b-5p | 1.684 | 0.752 | 0.277 | 0.007 | 0.193 |
