# Supplementary material for: Differential miRNA expression in B cells is associated with inter-individual differences in humoral immune response to measles vaccination
Source: PLoS One. 2018 Jan 30;13(1):e0191812. doi: 10.1371/journal.pone.0191812 (PMC5790242; doi:10.1371/journal.pone.0191812)
Supplement: S2 Table — (DOCX) [file pone.0191812.s002.docx]

**Table S2** miRNA expression in MV-stimulated CD4^+^ T cells irrespective of immune response status (i.e., overall analysis in all samples, q<0.2)

| **miRNA** | **FC** | **Log2FC** | **Std.Err.Log2FC** | **p-value** | **q-value** |
| --- | --- | --- | --- | --- | --- |
| hsa-miR-651-5p | 1.800 | 0.848 | 0.196 | 1.49E-05 | 0.0006 |
| hsa-miR-3614-5p | 5.443 | 2.444 | 0.575 | 2.09E-05 | 0.0006 |
| hsa-miR-21-5p | 1.776 | 0.829 | 0.197 | 2.58E-05 | 0.0006 |
| hsa-miR-3913-5p | 1.716 | 0.779 | 0.199 | 8.94E-05 | 0.002 |
| hsa-miR-199a-3p | 1.740 | 0.799 | 0.206 | 0.0001 | 0.002 |
| hsa-miR-106b-5p | 1.728 | 0.789 | 0.210 | 0.0002 | 0.002 |
| hsa-miR-126-3p | 1.660 | 0.731 | 0.194 | 0.0002 | 0.002 |
| hsa-let-7i-5p | 1.548 | 0.630 | 0.169 | 0.0002 | 0.002 |
| hsa-miR-598-3p | 1.866 | 0.900 | 0.242 | 0.0002 | 0.002 |
| hsa-miR-1307-5p | 1.790 | 0.840 | 0.232 | 0.0003 | 0.002 |
| hsa-miR-4658 | 2.351 | 1.233 | 0.356 | 0.001 | 0.003 |
| hsa-miR-5100 | 2.740 | 1.454 | 0.423 | 0.001 | 0.003 |
| hsa-miR-1248 | 1.436 | 0.522 | 0.152 | 0.001 | 0.003 |
| hsa-miR-142-3p | 1.586 | 0.665 | 0.197 | 0.001 | 0.004 |
| hsa-miR-25-5p | 1.627 | 0.703 | 0.210 | 0.001 | 0.004 |
| hsa-miR-92b-3p | 1.557 | 0.638 | 0.193 | 0.001 | 0.004 |
| hsa-miR-3202 | 1.586 | 0.665 | 0.203 | 0.001 | 0.005 |
| hsa-miR-4446-3p | 1.540 | 0.623 | 0.195 | 0.001 | 0.006 |
| hsa-miR-760 | 1.520 | 0.604 | 0.191 | 0.002 | 0.006 |
| hsa-miR-32-5p | 1.466 | 0.552 | 0.175 | 0.002 | 0.006 |
| hsa-miR-450a-5p | 2.150 | 1.104 | 0.351 | 0.002 | 0.006 |
| hsa-miR-4510 | 1.603 | 0.681 | 0.217 | 0.002 | 0.006 |
| hsa-miR-409-3p | 1.827 | 0.870 | 0.279 | 0.002 | 0.006 |
| hsa-miR-92a-1-5p | 1.733 | 0.793 | 0.261 | 0.002 | 0.007 |
| hsa-miR-7-5p | 1.428 | 0.514 | 0.169 | 0.002 | 0.007 |
| hsa-miR-659-5p | 1.492 | 0.577 | 0.190 | 0.002 | 0.007 |
| hsa-miR-5701 | 1.884 | 0.914 | 0.302 | 0.002 | 0.007 |
| hsa-miR-1827 | 1.426 | 0.512 | 0.169 | 0.002 | 0.007 |
| hsa-miR-223-5p | 1.588 | 0.667 | 0.222 | 0.003 | 0.007 |
| hsa-miR-3679-5p | 1.608 | 0.686 | 0.233 | 0.003 | 0.008 |
| hsa-miR-193b-5p | 1.546 | 0.628 | 0.215 | 0.004 | 0.009 |
| hsa-miR-3960 | 1.513 | 0.597 | 0.207 | 0.004 | 0.009 |
| hsa-miR-3607-3p | 2.027 | 1.020 | 0.357 | 0.004 | 0.010 |
| hsa-miR-1468-5p | 1.520 | 0.604 | 0.212 | 0.004 | 0.010 |
| hsa-miR-6868-3p | 1.599 | 0.677 | 0.240 | 0.005 | 0.010 |
| hsa-miR-378d | 1.428 | 0.514 | 0.183 | 0.005 | 0.010 |
| hsa-miR-210-3p | 1.463 | 0.549 | 0.197 | 0.005 | 0.011 |
| hsa-miR-27b-3p | 1.396 | 0.481 | 0.176 | 0.006 | 0.012 |
| hsa-miR-148b-5p | 1.453 | 0.539 | 0.198 | 0.006 | 0.012 |
| hsa-miR-212-5p | 1.923 | 0.943 | 0.347 | 0.007 | 0.012 |
| hsa-let-7b-5p | 1.486 | 0.572 | 0.213 | 0.007 | 0.013 |
| hsa-miR-1294 | 1.533 | 0.616 | 0.231 | 0.008 | 0.014 |
| hsa-miR-6130 | 1.380 | 0.465 | 0.176 | 0.008 | 0.014 |
| hsa-miR-629-5p | 1.452 | 0.538 | 0.206 | 0.009 | 0.015 |
| hsa-miR-206 | 1.463 | 0.548 | 0.210 | 0.009 | 0.015 |
| hsa-miR-27a-3p | 1.486 | 0.571 | 0.219 | 0.009 | 0.015 |
| hsa-miR-200a-3p | 1.455 | 0.541 | 0.209 | 0.010 | 0.016 |
| hsa-miR-499a-5p | 1.514 | 0.598 | 0.232 | 0.010 | 0.016 |
| hsa-miR-4448 | 1.805 | 0.852 | 0.332 | 0.010 | 0.016 |
| hsa-miR-7641 | 1.511 | 0.595 | 0.233 | 0.011 | 0.016 |
| hsa-miR-942-5p | 1.657 | 0.729 | 0.287 | 0.011 | 0.016 |
| hsa-miR-181a-5p | 1.423 | 0.509 | 0.200 | 0.011 | 0.016 |
| hsa-miR-652-3p | 1.543 | 0.626 | 0.249 | 0.012 | 0.016 |
| hsa-let-7a-5p | 1.454 | 0.540 | 0.215 | 0.012 | 0.016 |
| hsa-miR-4791 | 1.502 | 0.587 | 0.234 | 0.012 | 0.016 |
| hsa-miR-181b-5p | 1.414 | 0.500 | 0.200 | 0.012 | 0.016 |
| hsa-miR-92a-3p | 1.478 | 0.564 | 0.226 | 0.013 | 0.016 |
| hsa-miR-122-5p | 1.614 | 0.690 | 0.277 | 0.013 | 0.016 |
| hsa-miR-140-5p | 1.509 | 0.594 | 0.239 | 0.013 | 0.016 |
| hsa-miR-193a-5p | 1.562 | 0.644 | 0.259 | 0.013 | 0.016 |
| hsa-miR-125a-3p | 1.474 | 0.559 | 0.226 | 0.013 | 0.016 |
| hsa-miR-6501-5p | 1.788 | 0.838 | 0.344 | 0.015 | 0.018 |
| hsa-miR-92b-5p | 1.495 | 0.581 | 0.242 | 0.016 | 0.019 |
| hsa-let-7g-5p | 1.390 | 0.475 | 0.199 | 0.017 | 0.020 |
| hsa-let-7c-5p | 1.379 | 0.464 | 0.195 | 0.018 | 0.020 |
| hsa-miR-4488 | 1.848 | 0.886 | 0.376 | 0.018 | 0.020 |
| hsa-miR-30a-3p | 1.500 | 0.585 | 0.248 | 0.018 | 0.020 |
| hsa-miR-1292-5p | 1.564 | 0.645 | 0.274 | 0.019 | 0.021 |
| hsa-miR-1278 | 1.561 | 0.642 | 0.274 | 0.019 | 0.021 |
| hsa-miR-101-3p | 1.374 | 0.459 | 0.197 | 0.020 | 0.021 |
| hsa-miR-98-5p | 1.466 | 0.552 | 0.238 | 0.020 | 0.021 |
| hsa-miR-1246 | 1.591 | 0.670 | 0.289 | 0.021 | 0.021 |
| hsa-miR-320c | 1.362 | 0.445 | 0.193 | 0.021 | 0.021 |
| hsa-miR-3934-5p | 1.484 | 0.569 | 0.246 | 0.021 | 0.021 |
| hsa-miR-3605-3p | 1.504 | 0.589 | 0.255 | 0.021 | 0.021 |
| hsa-miR-221-3p | 1.397 | 0.483 | 0.209 | 0.021 | 0.021 |
| hsa-miR-574-5p | 1.555 | 0.637 | 0.279 | 0.022 | 0.021 |
| hsa-miR-664b-5p | 1.481 | 0.567 | 0.249 | 0.023 | 0.021 |
| hsa-miR-1290 | 1.598 | 0.677 | 0.297 | 0.023 | 0.021 |
| hsa-miR-425-3p | 1.346 | 0.429 | 0.189 | 0.023 | 0.021 |
| hsa-miR-30d-3p | 1.548 | 0.630 | 0.277 | 0.023 | 0.021 |
| hsa-miR-339-3p | 1.448 | 0.534 | 0.236 | 0.024 | 0.021 |
| hsa-miR-769-5p | 1.374 | 0.458 | 0.203 | 0.024 | 0.021 |
| hsa-miR-32-3p | 1.450 | 0.536 | 0.239 | 0.025 | 0.021 |
| hsa-miR-320d | 1.388 | 0.473 | 0.211 | 0.025 | 0.021 |
| hsa-miR-99b-3p | 1.447 | 0.533 | 0.238 | 0.025 | 0.021 |
| hsa-miR-25-3p | 1.394 | 0.480 | 0.214 | 0.025 | 0.021 |
| hsa-miR-4536-5p | 1.431 | 0.517 | 0.232 | 0.026 | 0.022 |
| hsa-miR-182-5p | 1.354 | 0.437 | 0.198 | 0.028 | 0.023 |
| hsa-miR-6803-3p | 1.590 | 0.669 | 0.306 | 0.029 | 0.024 |
| hsa-miR-766-5p | 1.375 | 0.459 | 0.211 | 0.030 | 0.024 |
| hsa-miR-6780a-5p | 1.499 | 0.584 | 0.270 | 0.031 | 0.025 |
| hsa-miR-125a-5p | 1.500 | 0.585 | 0.271 | 0.031 | 0.025 |
| hsa-miR-3653-3p | 1.424 | 0.509 | 0.236 | 0.031 | 0.025 |
| hsa-miR-423-5p | 1.373 | 0.458 | 0.213 | 0.032 | 0.025 |
| hsa-miR-30e-5p | 1.362 | 0.446 | 0.208 | 0.032 | 0.025 |
| hsa-miR-146b-3p | 1.358 | 0.442 | 0.206 | 0.032 | 0.025 |
| hsa-miR-330-5p | 1.338 | 0.420 | 0.197 | 0.033 | 0.025 |
| hsa-miR-320b | 1.380 | 0.465 | 0.219 | 0.033 | 0.025 |
| hsa-miR-342-5p | 1.377 | 0.462 | 0.217 | 0.034 | 0.025 |
| hsa-miR-150-3p | 1.412 | 0.498 | 0.236 | 0.035 | 0.026 |
| hsa-miR-1273h-3p | 1.385 | 0.470 | 0.224 | 0.035 | 0.026 |
| hsa-miR-4800-3p | 1.555 | 0.637 | 0.305 | 0.037 | 0.027 |
| hsa-miR-27a-5p | 1.312 | 0.392 | 0.188 | 0.037 | 0.027 |
| hsa-miR-642a-5p | 1.368 | 0.452 | 0.217 | 0.037 | 0.027 |
| hsa-miR-30e-3p | 1.375 | 0.459 | 0.221 | 0.038 | 0.027 |
| hsa-miR-6859-5p | 1.378 | 0.462 | 0.225 | 0.040 | 0.027 |
| hsa-miR-29a-3p | 1.299 | 0.377 | 0.184 | 0.040 | 0.027 |
| hsa-miR-3691-5p | 1.368 | 0.452 | 0.221 | 0.040 | 0.027 |
| hsa-let-7f-5p | 1.375 | 0.460 | 0.225 | 0.041 | 0.027 |
| hsa-miR-4508 | 1.754 | 0.811 | 0.397 | 0.041 | 0.027 |
| hsa-miR-4485-3p | 1.335 | 0.417 | 0.205 | 0.041 | 0.028 |
| hsa-miR-22-5p | 1.682 | 0.751 | 0.369 | 0.042 | 0.028 |
| hsa-miR-532-5p | 1.333 | 0.415 | 0.204 | 0.042 | 0.028 |
| hsa-miR-132-5p | 1.361 | 0.444 | 0.221 | 0.044 | 0.028 |
| hsa-miR-581 | 2.052 | 1.037 | 0.515 | 0.044 | 0.028 |
| hsa-miR-107 | 1.340 | 0.423 | 0.210 | 0.045 | 0.028 |
| hsa-miR-1273h-5p | 1.541 | 0.624 | 0.311 | 0.045 | 0.028 |
| hsa-miR-619-5p | 1.531 | 0.614 | 0.307 | 0.045 | 0.028 |
| hsa-miR-363-3p | 1.366 | 0.450 | 0.227 | 0.048 | 0.030 |
| hsa-miR-19a-3p | 1.488 | 0.574 | 0.290 | 0.048 | 0.030 |
| hsa-miR-190b | 1.311 | 0.391 | 0.198 | 0.048 | 0.030 |
| hsa-miR-6842-3p | 1.318 | 0.399 | 0.203 | 0.049 | 0.030 |
| hsa-miR-100-5p | 1.376 | 0.460 | 0.236 | 0.051 | 0.031 |
| hsa-miR-2110 | 1.398 | 0.484 | 0.248 | 0.051 | 0.031 |
| hsa-miR-4661-5p | 1.318 | 0.398 | 0.205 | 0.052 | 0.031 |
| hsa-miR-29c-3p | 1.480 | 0.565 | 0.293 | 0.054 | 0.032 |
| hsa-miR-10b-5p | 1.410 | 0.496 | 0.259 | 0.055 | 0.032 |
| hsa-miR-340-5p | 1.279 | 0.355 | 0.185 | 0.055 | 0.032 |
| hsa-miR-1285-3p | 1.397 | 0.483 | 0.253 | 0.057 | 0.033 |
| hsa-miR-378c | 1.312 | 0.391 | 0.206 | 0.057 | 0.033 |
| hsa-miR-3615 | 1.351 | 0.434 | 0.229 | 0.058 | 0.033 |
| hsa-miR-30b-3p | 1.439 | 0.525 | 0.277 | 0.058 | 0.033 |
| hsa-miR-215-5p | 1.195 | 0.257 | 0.136 | 0.059 | 0.033 |
| hsa-miR-361-3p | 1.271 | 0.346 | 0.186 | 0.062 | 0.034 |
| hsa-miR-4473 | 1.323 | 0.404 | 0.217 | 0.063 | 0.035 |
| hsa-miR-423-3p | 1.370 | 0.454 | 0.246 | 0.065 | 0.035 |
| hsa-miR-374a-5p | 1.327 | 0.408 | 0.222 | 0.066 | 0.035 |
| hsa-miR-7706 | 1.359 | 0.443 | 0.241 | 0.066 | 0.035 |
| hsa-miR-5193 | 1.344 | 0.427 | 0.232 | 0.066 | 0.035 |
| hsa-miR-378a-3p | 1.373 | 0.457 | 0.249 | 0.067 | 0.035 |
| hsa-miR-143-3p | 1.436 | 0.522 | 0.287 | 0.068 | 0.036 |
| hsa-miR-3656 | 1.790 | 0.840 | 0.462 | 0.069 | 0.036 |
| hsa-miR-320a | 1.432 | 0.518 | 0.286 | 0.070 | 0.036 |
| hsa-miR-1306-3p | 1.457 | 0.543 | 0.301 | 0.071 | 0.036 |
| hsa-miR-3148 | 1.410 | 0.495 | 0.274 | 0.071 | 0.036 |
| hsa-miR-3173-5p | 1.417 | 0.503 | 0.279 | 0.072 | 0.036 |
| hsa-miR-30c-1-3p | 1.299 | 0.377 | 0.210 | 0.072 | 0.036 |
| hsa-miR-181d-5p | 1.306 | 0.385 | 0.214 | 0.073 | 0.036 |
| hsa-miR-664a-5p | 1.385 | 0.470 | 0.262 | 0.073 | 0.036 |
| hsa-miR-486-3p | 1.375 | 0.460 | 0.257 | 0.073 | 0.036 |
| hsa-miR-6514-5p | 1.509 | 0.593 | 0.332 | 0.074 | 0.036 |
| hsa-miR-340-3p | 1.274 | 0.349 | 0.196 | 0.076 | 0.037 |
| hsa-miR-5091 | 1.442 | 0.529 | 0.299 | 0.077 | 0.037 |
| hsa-let-7d-3p | 1.375 | 0.460 | 0.260 | 0.077 | 0.037 |
| hsa-miR-374a-3p | 1.268 | 0.343 | 0.194 | 0.077 | 0.037 |
| hsa-miR-186-5p | 1.327 | 0.408 | 0.231 | 0.077 | 0.037 |
| hsa-miR-548q | 1.528 | 0.611 | 0.348 | 0.079 | 0.037 |
| hsa-miR-4649-5p | 1.516 | 0.601 | 0.342 | 0.079 | 0.037 |
| hsa-miR-6873-3p | 1.287 | 0.364 | 0.211 | 0.084 | 0.039 |
| hsa-miR-4443 | 1.423 | 0.509 | 0.296 | 0.085 | 0.039 |
| hsa-miR-128-3p | 1.325 | 0.406 | 0.236 | 0.085 | 0.039 |
| hsa-let-7d-5p | 1.373 | 0.457 | 0.271 | 0.092 | 0.042 |
| hsa-miR-877-5p | 1.306 | 0.385 | 0.230 | 0.094 | 0.042 |
| hsa-miR-6511a-3p | 1.573 | 0.653 | 0.391 | 0.094 | 0.042 |
| hsa-miR-363-5p | 1.381 | 0.465 | 0.278 | 0.094 | 0.042 |
| hsa-miR-194-5p | 1.345 | 0.428 | 0.256 | 0.095 | 0.043 |
| hsa-miR-106b-3p | 1.287 | 0.363 | 0.219 | 0.097 | 0.043 |
| hsa-miR-16-2-3p | 1.313 | 0.393 | 0.237 | 0.097 | 0.043 |
| hsa-miR-148a-3p | 1.266 | 0.340 | 0.206 | 0.098 | 0.043 |
| hsa-miR-20a-5p | 1.406 | 0.492 | 0.299 | 0.100 | 0.044 |
| hsa-miR-93-5p | 1.368 | 0.452 | 0.276 | 0.101 | 0.044 |
| hsa-miR-6515-5p | 1.351 | 0.434 | 0.266 | 0.103 | 0.044 |
| hsa-miR-296-3p | 1.301 | 0.379 | 0.234 | 0.104 | 0.045 |
| hsa-miR-330-3p | 1.329 | 0.410 | 0.255 | 0.107 | 0.045 |
| hsa-miR-16-5p | 1.360 | 0.443 | 0.276 | 0.108 | 0.045 |
| hsa-miR-99b-5p | 1.280 | 0.356 | 0.221 | 0.108 | 0.045 |
| hsa-miR-28-5p | 1.344 | 0.427 | 0.265 | 0.108 | 0.045 |
| hsa-miR-5096 | 1.459 | 0.545 | 0.340 | 0.109 | 0.045 |
| hsa-miR-543 | 1.564 | 0.645 | 0.403 | 0.110 | 0.045 |
| hsa-miR-22-3p | 1.306 | 0.385 | 0.241 | 0.111 | 0.046 |
| hsa-miR-31-5p | 1.318 | 0.399 | 0.250 | 0.111 | 0.046 |
| hsa-miR-4461 | 1.245 | 0.316 | 0.200 | 0.114 | 0.046 |
| hsa-miR-548o-3p | 1.285 | 0.362 | 0.229 | 0.115 | 0.046 |
| hsa-miR-130b-3p | 1.410 | 0.495 | 0.317 | 0.118 | 0.048 |
| hsa-miR-23b-5p | 1.347 | 0.430 | 0.276 | 0.119 | 0.048 |
| hsa-miR-26b-5p | 1.289 | 0.366 | 0.235 | 0.119 | 0.048 |
| hsa-miR-505-5p | 1.353 | 0.436 | 0.280 | 0.120 | 0.048 |
| hsa-miR-6855-5p | 1.338 | 0.420 | 0.272 | 0.122 | 0.048 |
| hsa-miR-1260b | 1.467 | 0.553 | 0.363 | 0.128 | 0.050 |
| hsa-miR-19b-3p | 1.311 | 0.391 | 0.257 | 0.129 | 0.050 |
| hsa-miR-5090 | 1.351 | 0.434 | 0.286 | 0.129 | 0.050 |
| hsa-miR-95-3p | 1.296 | 0.374 | 0.246 | 0.129 | 0.050 |
| hsa-miR-451a | 1.458 | 0.544 | 0.359 | 0.130 | 0.050 |
| hsa-miR-1307-3p | 1.315 | 0.395 | 0.262 | 0.132 | 0.050 |
| hsa-miR-142-5p | 1.301 | 0.380 | 0.254 | 0.135 | 0.051 |
| hsa-miR-501-3p | 1.323 | 0.404 | 0.271 | 0.135 | 0.051 |
| hsa-miR-7704 | 1.227 | 0.295 | 0.198 | 0.136 | 0.051 |
| hsa-miR-9-5p | 1.405 | 0.490 | 0.330 | 0.137 | 0.051 |
| hsa-miR-29b-3p | 1.204 | 0.268 | 0.181 | 0.139 | 0.052 |
| hsa-miR-3158-3p | 1.287 | 0.364 | 0.249 | 0.144 | 0.053 |
| hsa-miR-146a-5p | 1.319 | 0.400 | 0.276 | 0.148 | 0.055 |
| hsa-miR-185-5p | 1.330 | 0.412 | 0.286 | 0.150 | 0.055 |
| hsa-miR-6087 | 1.319 | 0.400 | 0.279 | 0.151 | 0.055 |
| hsa-miR-15b-5p | 1.331 | 0.413 | 0.289 | 0.154 | 0.056 |
| hsa-miR-4677-3p | 1.280 | 0.357 | 0.250 | 0.154 | 0.056 |
| hsa-miR-1255b-5p | 1.385 | 0.470 | 0.330 | 0.155 | 0.056 |
| hsa-miR-3179 | 1.494 | 0.579 | 0.407 | 0.155 | 0.056 |
| hsa-miR-10a-5p | 1.290 | 0.368 | 0.259 | 0.156 | 0.056 |
| hsa-miR-1260a | 1.463 | 0.549 | 0.389 | 0.159 | 0.056 |
| hsa-miR-24-3p | 1.295 | 0.373 | 0.268 | 0.164 | 0.058 |
| hsa-miR-1301-3p | 1.276 | 0.352 | 0.253 | 0.165 | 0.058 |
| hsa-miR-3653-5p | 1.302 | 0.380 | 0.274 | 0.166 | 0.058 |
| hsa-miR-28-3p | 1.302 | 0.381 | 0.275 | 0.166 | 0.058 |
| hsa-miR-4688 | 1.368 | 0.452 | 0.327 | 0.166 | 0.058 |
| hsa-miR-181a-3p | 1.242 | 0.313 | 0.231 | 0.174 | 0.060 |
| hsa-miR-484 | 1.309 | 0.389 | 0.286 | 0.175 | 0.060 |
| hsa-miR-200b-3p | 1.282 | 0.358 | 0.264 | 0.175 | 0.060 |
| hsa-miR-3605-5p | 1.325 | 0.406 | 0.299 | 0.175 | 0.060 |
| hsa-miR-103a-3p | 1.308 | 0.387 | 0.287 | 0.178 | 0.060 |
| hsa-miR-17-5p | 1.325 | 0.406 | 0.304 | 0.182 | 0.061 |
| hsa-miR-625-3p | 1.258 | 0.331 | 0.249 | 0.184 | 0.061 |
| hsa-miR-200c-3p | 1.300 | 0.379 | 0.285 | 0.184 | 0.061 |
| hsa-miR-155-5p | 1.300 | 0.379 | 0.285 | 0.185 | 0.061 |
| hsa-miR-3064-5p | 1.250 | 0.322 | 0.243 | 0.185 | 0.061 |
| hsa-miR-589-5p | 1.261 | 0.334 | 0.257 | 0.193 | 0.064 |
| hsa-miR-486-5p | 1.231 | 0.300 | 0.232 | 0.197 | 0.065 |
| hsa-miR-99a-5p | 1.209 | 0.274 | 0.215 | 0.203 | 0.066 |
| hsa-miR-130b-5p | 1.291 | 0.368 | 0.290 | 0.204 | 0.066 |
| hsa-miR-625-5p | 1.217 | 0.284 | 0.224 | 0.205 | 0.066 |
| hsa-miR-6769b-3p | 1.320 | 0.400 | 0.320 | 0.211 | 0.068 |
| hsa-miR-18a-3p | 1.295 | 0.373 | 0.300 | 0.213 | 0.068 |
| hsa-miR-576-3p | 1.265 | 0.340 | 0.273 | 0.214 | 0.069 |
| hsa-miR-744-5p | 1.272 | 0.347 | 0.280 | 0.215 | 0.069 |
| hsa-miR-125b-5p | 1.265 | 0.339 | 0.277 | 0.221 | 0.070 |
| hsa-miR-3138 | 1.324 | 0.405 | 0.334 | 0.226 | 0.072 |
| hsa-miR-20b-5p | 1.328 | 0.410 | 0.339 | 0.227 | 0.072 |
| hsa-miR-671-3p | 1.224 | 0.291 | 0.243 | 0.231 | 0.072 |
| hsa-miR-4326 | 1.321 | 0.402 | 0.339 | 0.236 | 0.074 |
| hsa-miR-628-3p | 1.276 | 0.352 | 0.299 | 0.239 | 0.074 |
| hsa-miR-185-3p | 1.282 | 0.359 | 0.305 | 0.240 | 0.074 |
| hsa-miR-26a-5p | 1.245 | 0.317 | 0.273 | 0.246 | 0.076 |
| hsa-miR-424-3p | 1.308 | 0.387 | 0.336 | 0.249 | 0.077 |
| hsa-miR-151a-3p | 1.226 | 0.294 | 0.259 | 0.256 | 0.078 |
| hsa-miR-335-3p | 1.254 | 0.326 | 0.292 | 0.264 | 0.080 |
| hsa-miR-6842-5p | 1.270 | 0.345 | 0.310 | 0.266 | 0.081 |
| hsa-miR-1255a | 1.219 | 0.286 | 0.260 | 0.272 | 0.082 |
| hsa-miR-140-3p | 1.187 | 0.247 | 0.231 | 0.286 | 0.086 |
| hsa-miR-1291 | 1.226 | 0.294 | 0.281 | 0.294 | 0.088 |
| hsa-miR-6786-3p | 1.303 | 0.382 | 0.366 | 0.296 | 0.088 |
| hsa-miR-6866-5p | 1.177 | 0.235 | 0.226 | 0.298 | 0.089 |
| hsa-let-7a-3p | 1.292 | 0.369 | 0.357 | 0.300 | 0.089 |
| hsa-miR-642a-3p | 1.231 | 0.300 | 0.295 | 0.309 | 0.091 |
| hsa-miR-502-3p | 1.270 | 0.344 | 0.339 | 0.310 | 0.091 |
| hsa-miR-328-3p | 1.229 | 0.297 | 0.295 | 0.314 | 0.091 |
| hsa-miR-421 | 1.300 | 0.378 | 0.377 | 0.315 | 0.091 |
| hsa-miR-335-5p | 1.361 | 0.445 | 0.443 | 0.315 | 0.091 |
| hsa-miR-192-5p | 1.187 | 0.247 | 0.246 | 0.316 | 0.091 |
| hsa-miR-146b-5p | 1.230 | 0.298 | 0.298 | 0.317 | 0.091 |
| hsa-miR-4685-3p | 1.260 | 0.333 | 0.337 | 0.322 | 0.092 |
| hsa-miR-6747-3p | 1.219 | 0.286 | 0.290 | 0.324 | 0.093 |
| hsa-miR-152-3p | 1.197 | 0.259 | 0.263 | 0.325 | 0.093 |
| hsa-miR-221-5p | 1.219 | 0.286 | 0.295 | 0.332 | 0.094 |
| hsa-miR-365a-5p | 1.191 | 0.252 | 0.263 | 0.337 | 0.095 |
| hsa-miR-148a-5p | 1.198 | 0.261 | 0.274 | 0.341 | 0.096 |
| hsa-miR-425-5p | 1.198 | 0.261 | 0.276 | 0.344 | 0.096 |
| hsa-miR-29c-5p | 1.171 | 0.228 | 0.248 | 0.358 | 0.100 |
| hsa-miR-361-5p | 1.195 | 0.257 | 0.284 | 0.365 | 0.102 |
| hsa-miR-874-3p | 0.741 | -0.433 | 0.484 | 0.371 | 0.103 |
| hsa-miR-23a-5p | 1.162 | 0.216 | 0.249 | 0.386 | 0.107 |
| hsa-miR-181a-2-3p | 1.127 | 0.172 | 0.201 | 0.392 | 0.108 |
| hsa-miR-6741-3p | 1.213 | 0.279 | 0.328 | 0.396 | 0.109 |
| hsa-miR-23a-3p | 0.849 | -0.236 | 0.281 | 0.401 | 0.110 |
| hsa-miR-30b-5p | 1.160 | 0.214 | 0.256 | 0.405 | 0.110 |
| hsa-miR-4301 | 1.272 | 0.347 | 0.422 | 0.412 | 0.112 |
| hsa-miR-509-3-5p | 0.897 | -0.157 | 0.202 | 0.438 | 0.118 |
| hsa-miR-4647 | 1.232 | 0.301 | 0.392 | 0.443 | 0.119 |
| hsa-miR-6741-5p | 1.214 | 0.280 | 0.368 | 0.447 | 0.120 |
| hsa-miR-24-2-5p | 1.166 | 0.221 | 0.295 | 0.453 | 0.121 |
| hsa-miR-3609 | 1.136 | 0.183 | 0.250 | 0.463 | 0.124 |
| hsa-miR-183-5p | 1.126 | 0.171 | 0.238 | 0.472 | 0.125 |
| hsa-miR-30d-5p | 1.146 | 0.197 | 0.287 | 0.492 | 0.130 |
| hsa-miR-378i | 1.159 | 0.213 | 0.321 | 0.507 | 0.134 |
| hsa-miR-222-3p | 1.126 | 0.171 | 0.260 | 0.511 | 0.134 |
| hsa-miR-941 | 1.112 | 0.153 | 0.235 | 0.516 | 0.135 |
| hsa-miR-219a-1-3p | 1.102 | 0.140 | 0.216 | 0.517 | 0.135 |
| hsa-miR-1273g-3p | 1.105 | 0.144 | 0.222 | 0.518 | 0.135 |
| hsa-miR-30a-5p | 1.123 | 0.167 | 0.260 | 0.519 | 0.135 |
| hsa-miR-195-3p | 1.135 | 0.183 | 0.285 | 0.521 | 0.135 |
| hsa-miR-203a-3p | 1.258 | 0.331 | 0.519 | 0.524 | 0.135 |
| hsa-miR-3177-3p | 1.140 | 0.190 | 0.300 | 0.528 | 0.135 |
| hsa-miR-191-5p | 1.171 | 0.228 | 0.364 | 0.531 | 0.136 |
| hsa-miR-500a-3p | 1.144 | 0.195 | 0.317 | 0.539 | 0.137 |
| hsa-miR-125b-2-3p | 1.147 | 0.198 | 0.323 | 0.540 | 0.137 |
| hsa-miR-151a-5p | 1.116 | 0.158 | 0.264 | 0.549 | 0.139 |
| hsa-miR-196b-5p | 1.157 | 0.210 | 0.362 | 0.561 | 0.141 |
| hsa-miR-4449 | 1.124 | 0.168 | 0.294 | 0.567 | 0.142 |
| hsa-miR-194-3p | 1.117 | 0.160 | 0.283 | 0.573 | 0.143 |
| hsa-let-7b-3p | 1.171 | 0.227 | 0.403 | 0.573 | 0.143 |
| hsa-miR-509-3p | 1.088 | 0.122 | 0.219 | 0.579 | 0.144 |
| hsa-miR-148b-3p | 1.132 | 0.179 | 0.324 | 0.581 | 0.144 |
| hsa-miR-331-3p | 1.140 | 0.189 | 0.348 | 0.588 | 0.145 |
| hsa-miR-331-5p | 1.141 | 0.190 | 0.355 | 0.593 | 0.146 |
| hsa-miR-151b | 1.104 | 0.143 | 0.279 | 0.609 | 0.150 |
| hsa-miR-1275 | 1.126 | 0.172 | 0.340 | 0.614 | 0.150 |
| hsa-miR-505-3p | 0.880 | -0.184 | 0.370 | 0.618 | 0.151 |
| hsa-miR-582-3p | 1.099 | 0.137 | 0.278 | 0.623 | 0.151 |
| hsa-miR-1299 | 1.089 | 0.123 | 0.261 | 0.638 | 0.155 |
| hsa-miR-378f | 1.087 | 0.121 | 0.260 | 0.642 | 0.155 |
| hsa-let-7e-5p | 0.743 | -0.429 | 0.968 | 0.658 | 0.158 |
| hsa-miR-5009-5p | 1.072 | 0.100 | 0.253 | 0.693 | 0.166 |
| hsa-miR-197-3p | 1.091 | 0.125 | 0.327 | 0.702 | 0.168 |
| hsa-miR-432-5p | 1.113 | 0.154 | 0.416 | 0.711 | 0.169 |
| hsa-miR-139-5p | 0.916 | -0.127 | 0.342 | 0.711 | 0.169 |
| hsa-miR-664a-3p | 0.927 | -0.109 | 0.298 | 0.715 | 0.169 |
| hsa-miR-1-3p | 1.107 | 0.146 | 0.403 | 0.716 | 0.169 |
| hsa-miR-375 | 1.095 | 0.130 | 0.382 | 0.733 | 0.173 |
| hsa-miR-576-5p | 0.955 | -0.066 | 0.225 | 0.768 | 0.180 |
| hsa-miR-508-3p | 1.052 | 0.073 | 0.252 | 0.773 | 0.181 |
| hsa-miR-23b-3p | 0.951 | -0.073 | 0.258 | 0.778 | 0.181 |
| hsa-miR-374b-5p | 1.074 | 0.103 | 0.375 | 0.783 | 0.182 |
| hsa-miR-1271-5p | 1.057 | 0.080 | 0.301 | 0.789 | 0.183 |
| hsa-miR-106a-5p | 1.080 | 0.111 | 0.452 | 0.806 | 0.186 |
| hsa-miR-30c-5p | 1.059 | 0.083 | 0.356 | 0.816 | 0.188 |
| hsa-miR-150-5p | 1.045 | 0.064 | 0.278 | 0.818 | 0.188 |
| hsa-miR-548e-3p | 0.955 | -0.066 | 0.307 | 0.830 | 0.190 |
| hsa-miR-4520-3p | 1.059 | 0.083 | 0.430 | 0.846 | 0.193 |
| hsa-miR-26a-2-3p | 1.049 | 0.070 | 0.374 | 0.852 | 0.194 |
| hsa-miR-671-5p | 0.960 | -0.059 | 0.334 | 0.860 | 0.195 |
