# Supplementary material for: Differential miRNA expression in B cells is associated with inter-individual differences in humoral immune response to measles vaccination
Source: PLoS One. 2018 Jan 30;13(1):e0191812. doi: 10.1371/journal.pone.0191812 (PMC5790242; doi:10.1371/journal.pone.0191812)
Supplement: S3 Table — (DOCX) [file pone.0191812.s003.docx]

**Table S3** Pathways and biological processes differentially regulated upon stimulation with MV in B cells or CD4^+^ T cells (overall analysis in all samples)

| **Pathway** | **P-value** | **#Genes** | **#miRNAs** |
| --- | --- | --- | --- |
| **In B cells:** |  |  |  |
| Cellular nitrogen compound metabolic process | <1.0E-325 | 999 | 6 |
| Neurotrophin TRK receptor signaling pathway | <1.0E-325 | 63 | 4 |
| Fc receptor signaling pathway | <1.0E-325 | 51 | 4 |
| Nucleic acid binding transcription factor activity | <1.0E-325 | 225 | 4 |
| Protein binding transcription factor activity | <1.0E-325 | 121 | 3 |
| Epidermal growth factor receptor signaling pathway | 3.4E-13 | 59 | 4 |
| Phosphatidylinositol-mediated signaling | 1.79E-07 | 39 | 3 |
| Fibroblast growth factor receptor signaling pathway | 1.11E-06 | 47 | 4 |
| Extracellular matrix (ECM)-receptor interaction | 1.24E-06 | 7 | 3 |
| TGF-beta signaling pathway | 4.72E-06 | 32 | 3 |
| Transcription initiation from RNA polymerase II promoter | 7.25E-06 | 50 | 3 |
| Viral process | 0.00004 | 71 | 2 |
| Cell-cell signaling | 0.00017 | 107 | 2 |
| Signaling pathways regulating pluripotency of stem cells | 0.00061 | 46 | 2 |
| Positive regulation of transcription from RNA polymerase II promoter | 0.00145 | 223 | 2 |
| Cytoskeletal protein binding | 0.00182 | 114 | 2 |
| Cellular lipid metabolic process | 0.00460 | 28 | 2 |
| Transcription from RNA polymerase II promoter | 0.00493 | 109 | 2 |
| Toll-like receptor 10 signaling pathway | 0.00735 | 11 | 2 |
| Biosynthesis of unsaturated fatty acids | 0.00742 | 2 | 1 |
| Cell junction assembly | 0.00897 | 19 | 2 |
| Toll-like receptor 9 signaling pathway | 0.01031 | 12 | 2 |
| Toll-like receptor TLR1:TLR2 signaling pathway | 0.01117 | 11 | 2 |
| Toll-like receptor TLR6:TLR2 signaling pathway | 0.01117 | 11 | 2 |
| Transcription factor binding | 0.01442 | 66 | 1 |
| Toll-like receptor 5 signaling pathway | 0.01689 | 5 | 1 |
| Cell death | 0.03738 | 129 | 2 |
| **In CD4^+^ T cells:** |  |  |  |
| Cellular nitrogen compound metabolic process | <1.0E-325 | 829 | 8 |
| Neurotrophin TRK receptor signaling pathway | 1.48E-12 | 62 | 5 |
| Nucleic acid binding transcription factor activity | 6.8E-10 | 141 | 4 |
| Extracellular matrix (ECM)-receptor interaction | 1.46E-09 | 11 | 2 |
| Fc-receptor signaling pathway | 8.83E-09 | 39 | 5 |
| Epidermal growth factor receptor signaling pathway | 0.00004 | 40 | 4 |
| Protein binding transcription factor activity | 0.00125 | 73 | 3 |
| Viral process | 0.01484 | 53 | 3 |
| Glycosaminoglycan biosynthesis - chondroitin sulfate / dermatan sulfate | 0.01693 | 6 | 3 |
| Fibroblast growth factor receptor signaling pathway | 0.02499 | 24 | 2 |
| TGF-beta signaling pathway | 0.03742 | 18 | 2 |
