# Supplementary material for: Differential miRNA expression in B cells is associated with inter-individual differences in humoral immune response to measles vaccination
Source: PLoS One. 2018 Jan 30;13(1):e0191812. doi: 10.1371/journal.pone.0191812 (PMC5790242; doi:10.1371/journal.pone.0191812)
Supplement: S4 Table — (DOCX) [file pone.0191812.s004.docx]

**Table S4** CD4^+^ T cell-specific miRNA expression differences between high and low antibody responders to measles vaccination (no significant differences were noted, top 10 miRNAs are listed)

| **miRNA** | **FC** | **Log2FC** | **Std.Err.Log2FC** | **p-value** | **q-value** |
| --- | --- | --- | --- | --- | --- |
| hsa-miR-3656 | 0.331 | -1.596 | 0.494 | 0.001 | 0.418 |
| hsa-miR-1255b-5p | 0.353 | -1.501 | 0.498 | 0.003 | 0.436 |
| hsa-miR-4791 | 0.514 | -0.961 | 0.372 | 0.010 | 0.997 |
| hsa-miR-4488 | 0.402 | -1.316 | 0.564 | 0.020 | 0.997 |
| hsa-miR-6087 | 0.448 | -1.158 | 0.500 | 0.021 | 0.997 |
| hsa-let-7e-5p | 6.287 | 2.652 | 1.189 | 0.026 | 0.997 |
| hsa-miR-3614-5p | 0.221 | -2.180 | 1.027 | 0.034 | 0.997 |
| hsa-miR-3960 | 0.627 | -0.674 | 0.342 | 0.049 | 0.997 |
| hsa-miR-4508 | 0.452 | -1.145 | 0.595 | 0.054 | 0.997 |
| hsa-miR-378c | 0.614 | -0.704 | 0.375 | 0.060 | 0.997 |
